# A novel bovine leukemia virus peptide vaccine targeting susceptible cattle-Production by 3-D modelling and nanotechnology

**DOI:** 10.1186/1742-4690-12-S1-P48

**Published:** 2015-08-28

**Authors:** Yoko Aida, Jiyun Kim, Noriaki Okimoto, Junya Yamagishi, Seiichi Tada, Yoshihiro Ito, Shin-nosuke Takeshima

**Affiliations:** 1 Viral Infectious Diseases Unit, RIKEN, Wako, Saitama, Japan; 2 Computational Biology Research, Core, Quantitative Biology Center (QBiC), RIKEN, Kobe, Japan; 3 Nano Medical Engineering Laboratory, RIKEN, Wako, Saitama, Japan

## 

Bovine leukemia virus (BLV), a retrovirus related to human T-cell leukemia virus types 1 and 2 (HTLV-1 and -2), causes enzootic bovine leukosis (EBL), a disease characterized by a much extended course that often involves persistent lymphocytosis and culminates in B-cell lymphoma the future. Our previously study demonstrated that cattle which had bovine major histocompatibility complex (BoLA)-DRB3*1601/*1601 genotype tend to onset EBL and indicates high proviral load. One of the major problems to develop the BLV vaccine is unstable effect of the vaccine because of the individual difference for disease susceptibility, we here tried to develop the BLV vaccine for disease susceptible cattle. Firstly, we determined the Th1 epitope of BLV antigens against disease susceptible cattle using a total 118 kind of peptides corresponding to GAG p15, p24 and p12, and ENV gp51 and gp30. Furthermore, we performed the optimization of our determined Th1 epitopes by antigen peptide modeling using in silico screening to resolve low affinity binding between peptide and susceptible BoLA DR molecule. Moreover, to resolve low stability of peptide and induce effectively Th1 immunity, we capsulated our optimized peptides, p12-4R1 and gp51R1, by Carbonate apatite (CO3Ap) because out of three kinds of nano-particles, such as CO3Ap, Cholesterol-modified gelatin and MGlu-HPG-Liposome, CO3Ap most strongly induced in all of Dendritic cell incorporation, antibody production and cellular immunity. Finally, we demonstrated the induction of antigen-specific cell-mediated immune response in mice by both of subcutaneous vaccination and intradermal vaccination of p12-4R1/gp51R1 peptide-conjugated CO3Ap. Collectively, we succeeded to produce a novel peptide vaccine targeting disease susceptible cattle which can induce antigen-specific cell-mediated immune response in vivo.

